# The TRPV4 Agonist GSK1016790A Regulates the Membrane Expression of TRPV4 Channels

**DOI:** 10.3389/fphar.2019.00006

**Published:** 2019-01-23

**Authors:** Sara Baratchi, Peter Keov, William G. Darby, Austin Lai, Khashayar Khoshmanesh, Peter Thurgood, Parisa Vahidi, Karin Ejendal, Peter McIntyre

**Affiliations:** ^1^School of Health and Biomedical Sciences, RMIT University, Melbourne, VIC, Australia; ^2^Molecular Pharmacology Division, Victor Chang Cardiac Research Institute, Darlinghurst, NSW, Australia; ^3^St Vincent's Clinical School, University of New South Wales, Darlinghurst, NSW, Australia; ^4^School of Engineering, RMIT University, Melbourne, VIC, Australia; ^5^Weldon School of Biomedical Engineering, Purdue University, West Lafayette, IN, United States

**Keywords:** TRPV4, membrane trafficking, endothelial cells, GSK1016790A, calcium

## Abstract

TRPV4 is a non-selective cation channel that tunes the function of different tissues including the vascular endothelium, lung, chondrocytes, and neurons. GSK1016790A is the selective and potent agonist of TRPV4 and a pharmacological tool that is used to study the TRPV4 physiological function *in vitro* and *in vivo*. It remains unknown how the sensitivity of TRPV4 to this agonist is regulated. The spatial and temporal dynamics of receptors are the major determinants of cellular responses to stimuli. Membrane translocation has been shown to control the response of several members of the transient receptor potential (TRP) family of ion channels to different stimuli. Here, we show that TRPV4 stimulation with GSK1016790A caused an increase in [Ca^2+^]_i_ that is stable for a few minutes. Single molecule analysis of TRPV4 channels showed that the density of TRPV4 at the plasma membrane is controlled through two modes of membrane trafficking, complete, and partial vesicular fusion. Further, we show that the density of TRPV4 at the plasma membrane decreased within 20 min, as they translocate to the recycling endosomes and that the surface density is dependent on the release of calcium from the intracellular stores and is controlled via a PI3K, PKC, and RhoA signaling pathway.

## Introduction

Transient receptor potential vanilloid sub type 4 (TRPV4) is a non-selective multifunctional cation channel that is involved in different physiological function and also pathologies (Darby et al., 2016; White et al., 2016; Rajasekhar et al., 2017). TRPV4 channels can be stimulated by hypotonic stress, shear stress, moderate heat, phorbol ester (4α-PDD), and arachidonic acid metabolites, and such stimulation opens TRPV4 channels to increase calcium and sodium influx and consequently increase cytosolic intracellular calcium ([Ca^2+^]_i_) and sodium (Darby et al., 2016; Martinac and Poole, 2018). All ion channels must span a membrane which supports the appropriate electrochemical gradient to function. Thus, regulation of the trafficking to and from the plasma membrane as well as the spatial and temporal distribution of ion channels is essential for their activity and downstream cellular functions (Planells-Cases and Ferrer-Montiel, 2007). The surface expression of channels is dependent on vesicular fusion via trafficking to membrane, while endocytosis controls internalization. Although little information is available on the trafficking of transient receptor potential (TRP) channels to and from the plasma membrane, this is important for controlling channel function (Baratchi et al., 2017a). Several studies on the TRPV family members have shown that trafficking into to the plasma membrane plays an important role in the channel responses to different stimuli (Baratchi et al., 2016). For example, Insulin-like growth factor (IGF) stimulation is reported to induce the insertion of TRPV2 into the plasma membrane (Boels et al., 2001), inflammatory mediators control the regulated membrane trafficking of TRPV1(Van Buren et al., 2005), and shear stress mediates membrane trafficking of TRPV4 channels (Baratchi et al., 2016).

The signaling pathways that regulate translocation of TRPV4 in response to activating modalities are not well studied. Our previous study on the effect of shear stress on TRPV4 channel activity showed that it induces rapid membrane trafficking of TRPV4 channels to the cell membrane, and sensitized the response of TRPV4 to its selective agonist GSK1016790A (Baratchi et al., 2014, 2016, 2017b).

GSK1016790A is the potent and selective agonist of TRPV4 that can activate TRPV4 in different cell types (Darby et al., 2016). The ineffectiveness of 4αPDD in contrary to the GSK1016790A likely reflects the low potency of this agonist (Thorneloe et al., 2008). A recent study showed that GSK1016790A stimulation of endothelial cells inhibits the adhesion of monocytes to the TNF-α stimulated endothelial cells, and reduces atherosclerotic plaque formation in the relevant animal model (Xu et al., 2016). Further, it has been shown that stimulation with GSK1016790A caused the down-regulation of TRPV4 channels from the plasma membrane after 30 min (Jin et al., 2011), and the majority of TRPV4 channels at the plasma membrane are silent (Sullivan et al., 2012). Maximal stimulation with GSK1016790A increased the number of sites with localized [Ca^2+^]_i_ which is postulated to represent the recruitment of the new channels to the plasma membrane. However, the mechanism of TRPV4 response to GSK1016790A is not very well known.

Here, using the combination of Total Internal Reflectance Fluorescence (TIRF) microscopy, Ca^2+^ imaging, and Bioluminescence Resonance Energy Transfer (BRET) assay, we have investigated the effect of GSK1016790A on the trafficking of TRPV4 channels and identified the signaling pathway that controls such trafficking. Further, we elucidate the importance of this trafficking on global changes in [Ca^2+^]_i_ following GSK1016790A stimulation.

## Materials and Methods

### Compounds and Buffers

For the Ca^2+^ imaging experiments, cells were immersed in standard Hanks' balanced salt solution (HBSS) containing: 140 mmol/L NaCl, 5 mmol/L KCl, 10 mmol/L HEPES, 11 mmol/L D-glucose, 1 mmol/L MgCl_2_, 2 mmol/L CaCl_2_, and 2 mmol/L probenecid, adjusted to pH = 7.4 (Alabi and Tsien, 2013). To remove calcium from the buffer, the CaCl_2_ in HBSS was replaced with 2 mmol/L ethylene glycol tetraacetic acid (EGTA).

GSK1016790A (TRPV4-selective agonist), brefeldin A (an inhibitor of intracellular protein transport), cytochalasin D (an inhibitor of actin polymerization), and GSK690693 (Akt inhibitor) were purchased from Sigma-Aldrich (St. Louis, MO). HC067047 (TRPV4-selective antagonist) and NSC23766 (Rac1 inhibitor) were purchased from Santa Cruz Biotechnology (Santa Cruz, CA). Pipstop2 (Clathrin inhibitor) was purchased from Abcam. Cpd 22 (ILK inhibitor) was obtained from Calbiochem.

All drugs were dissolved according to the supplier's instruction (0.1% v/v dH_2_O or 0.1% v/v DMSO) and diluted in HBSS prior to experimentation, and corresponding vehicles (0.1% v/v dH_2_O or 0.1% v/v DMSO) were used as controls.

### Cell Culture, Molecular Biology and Gene Transfection

Human Umbilical Vein Endothelial Cells (HUVECs) were grown in EGMTM-2 media supplemented with SingleQuots^®^ (Lonza, Walkersville MD, USA). RhoA, RhoA (T19N), CDC42, CDC42 (T17N), Rac1, and Rac1 (T17N) cloned in vector pcDNA3.1 were obtained from cDNA Resource Center, University of Missouri-Rolla, MO. HEK293 T-Rex cell lines (Invitrogen, Carlsbad, CA, USA) stably expressing TRPV4 (TRPV4-Venus) were generated as described in Poole et al. (2013) and Baratchi et al. (2016) and grown in Dulbecco's Modified Eagle's medium (Thermo Fisher, Waltham, MA, USA) supplemented with 10% fetal bovine serum, hygromycin (50 μg/ml), and blasticidin (5 μg/ml).

### Calcium Imaging

For calcium imaging experiments, media was removed, and cells were washed with HBSS, then loaded with calcium-sensitive dyes in HBSS. Dyes were then washed off, and cells were treated with inhibitor buffer or corresponding vehicle controls, cells were either imaged on a plate reading fluorimeter (Flex station III, Molecular devices, San Jose, CA, USA) or using confocal microscopy (Baratchi et al., 2014). Plate reading experiments utilized FURA-2AM (2.5 μM) with pluronic acid (0.01% w/v) in HBSS, ex/em: 340/520 nm (Ca^2+^ bound) 380/520 nm (Ca^2+^ unbound) data represented at a ratio of 340/380 nm (Ca^2+^ bound/Ca^2+^ unbound). Microscopy experiments utilized FLUO-4AM (1 μM), ex/em: 450/520 nm, data represented as F_1_/F_0_, the ratio of fluorescence compared to fluorescence at the zero time point. Cells were imaged for baseline [Ca^2+^]_i_ then agonists were administered at the points indicated on the graphs.

### Bioluminescence Resonance Energy Transfer (BRET) Analysis of TRPV4 Association With Kras and Rab11

HEK293 cells stably expressing TRPV4-Venus were seeded on white, poly-L-lysine coated 96-well plate the day before, and transiently transfected with Rab11-Rluc8, Kras-Rluc8, or TRPV4-Rluc8 (0.5 μg/ well) using effectene (QIAGEN). Rab11-Rluc8 and Kras-Rluc8 were from Nevin A Lambert (Augusta University, Georgia, USA). At 48 h post-transfection, TRPV4 expression was induced using 0.1 μg/ml of tetracycline for 16 h. Before the BRET assay, the medium was replaced with HBSS 30 min before the assay. Cells were loaded with coelenterazine H (Nanolight Tech.) (5 μM), RLuc8 luminescence (480 nm) and Venus fluorescence (530 nm) were measured for a 2–3 min to establish a baseline, and at different times after addition of 100 nM GSK1016790A, using a CLARIOstar microplate reader (BMG LABTECH).

### Biotinylation of Surface Proteins

HUVECs were grown in a 24-well plate to confluence, incubated with inhibitors or the appropriate controls for at least 30 min. After TRPV4 stimulation with GSK101, EZ-Link Sulfo-NHS-SS linked biotin (0.5 mg/ml) was added in PBS, and incubated on ice for 30 min at 4°C. Following stimulation, the reaction was quenched with 50 mmol/L glycine and excess biotin was removed with PBS. Lysates were prepared in standard lysis buffer (1 mmol/L EDTA, 1 mmol/L EGTA, 30 mmol/L NaCl, 50 mmol/L Na2H2PO4, 2 mmol/L PMSF, Halt Protease Inhibitor Cocktail (Pierce), and 1% Triton-X-100). Samples were normalized for the protein concentrations and incubated with streptavidin beads overnight at 4°C, and the SDS page and Western Blotting was performed as reported previously (Baratchi et al., 2016).

### TIRF Microscopy and Image Analysis

Image acquisition, processing, and analysis was performed as reported before (Baratchi et al., 2016, 2017b). Briefly, for TIRFM we used a fully motorized Nikon Ti-E inverted microscope equipped with a CFI-APO 100×, 1.49 numerical aperture TIRF objective. For illumination of the Venus tagged TRPV4 channels, a coherent sapphire laser (488, Coherent Inc.) was used. The laser was focused using the back focal plane before each experiment. Images were acquired at 10 Hz using an iXonEM back-illuminated EMCCD camera (Andor, Belfast, UK). Live cell experiments were performed at 37°C using the Okolab objective heater (OKOLAB USA Inc., Burlingame, CA). Images shown represent raw data with simple background subtraction of the average blank field intensity. Intensity values were corrected for photobleaching during image collection.

### Statistical Analysis

Results are reported as the mean ± SEM. We used the unpaired *t*-test to examine differences between two groups, and ANOVA and a Dunnett's *post-hoc* test to examine differences between multiple groups. *P* < 0.05 was considered significant.

## Results

### TRPV4 Response to GSK1016790A Stimulation Reduces the Plasma Membrane Density of TRPV4 Channels

**Figure 1:**
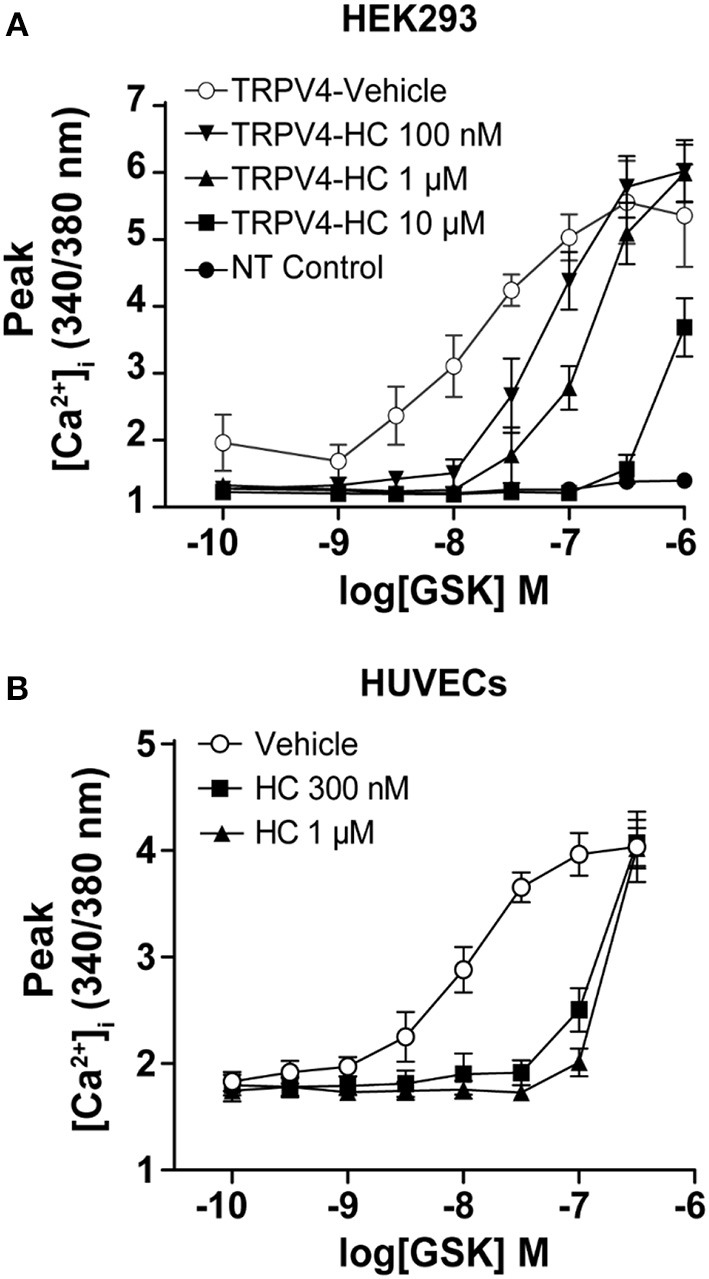
GSK1016790A induced Ca^2+^ influx in endothelial cells and HEK293 cells stably expressing TRPV4. Ca^2+^ imaging showing that treatment with GSK1016790A induced Ca^2+^ influx in **(A)** HEK293-TRPV4 cell line and **(B)** HUVECs and inhibition of TRPV4 with HC067047 abolished the GSK1016790A induced Ca^2+^ influx in both cell types. Data are representative of 3–4 independent experiments and are presented as mean ± SEM. NT in **(A)** represents non-transfected parental HEK293 cells.

To study the effect of TRPV4 selective agonist GSK1016790A, we used HEK293 T-REx cells stably expressing TRPV4 or human umbilical vein endothelial cells (HUVECs). First, to test the specificity of GSK1016790A, we used non-transfected HEK293 cells or treated cells with a selective antagonist of TRPV4, HC067047(Vincent and Duncton, 2011). For both conditions, GSK1016790A failed to increase the [Ca^2+^]_i_ (Figure 1A).

As reported previously, we found that treatment of HUVECs and TRPV4-HEK293 cells with GSK1016790A lead to the activation of the TRPV4 and an increase in [Ca^2+^]_i_ within the first-minute post-treatment (Figure 1B, Supplementary Image 1, Supplementary Video 1).

**Figure 2:**
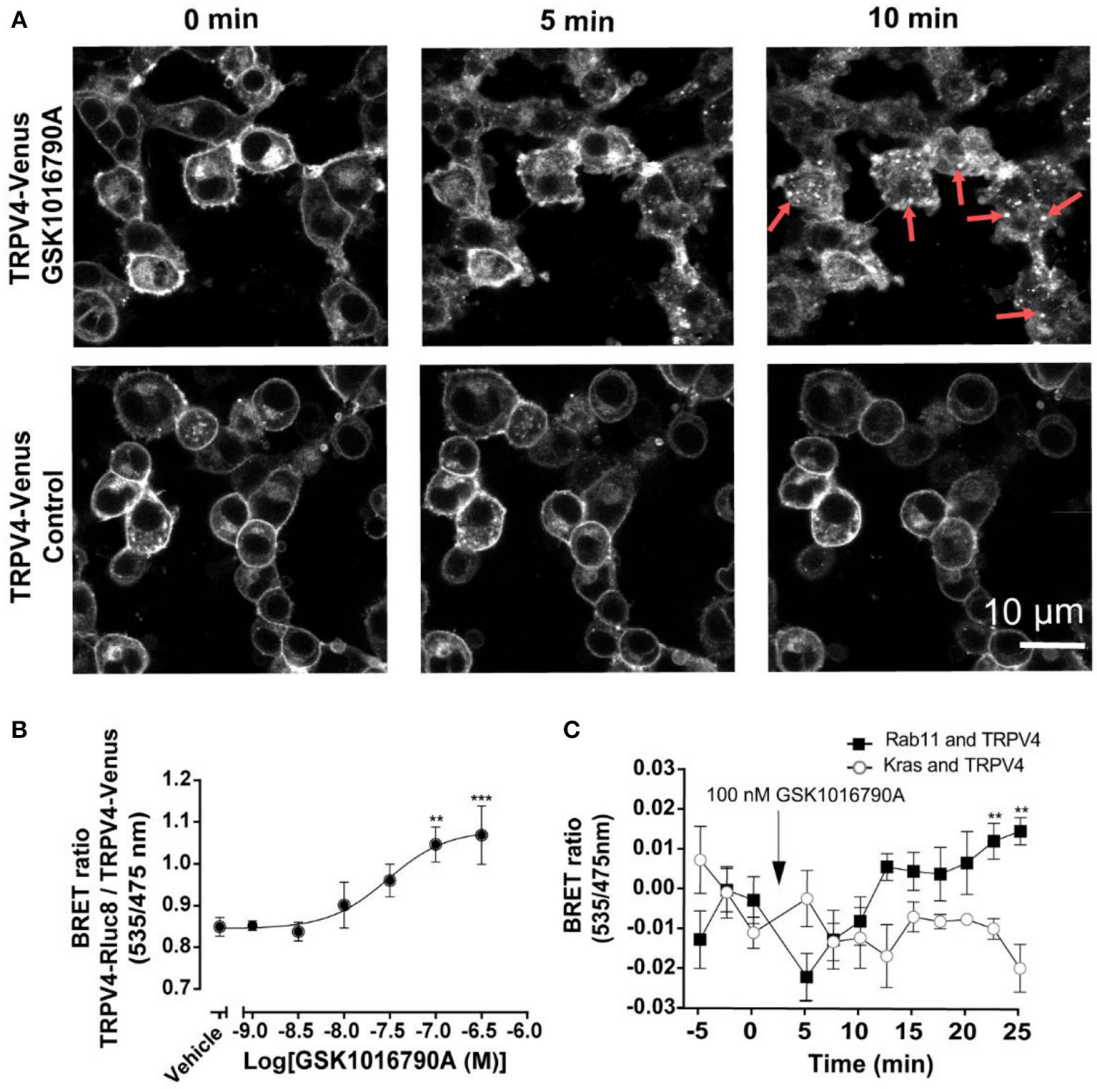
GSK1016790A induced the endocytosis and complexation of TRPV4 into recycling endosomes. **(A)** Confocal microscopy of HEK293 cells stably expressing TRPV4-Venus, showing the stimulation of TRPV4 with 100 nM GSK1016790A induced the internalization and complexation of TRPV4 channels. **(B)** Stimulation of TRPV4 with GSK1016790A increased the BRET signals between tagged TRPV4 channels. **(C)** BRET assay showing that TRPV4 stimulation with GSK1016790A increased the interaction of TRPV4 channels with Rab11 while reducing the interaction with Kras. Data in **(B,C)** represent the mean ± SEM. of four independent experiments performed in duplicate. ^**^p < 0.01 and ^***^p < 0.001.

To measure the effect of GSK1016790A on the translocation of TRPV4 channels, initially, we used TRPV4 channels tagged with Venus stably expressing in HEK293 cells. As shown in Figure 2A, after GSK1016790A stimulation, TRPV4 channels formed vesicular structures in the cytoplasm that was easily detectable after 5, and 10 min post-GSK1016790A that were absent in the untreated control group imaged at the same time point (Figure 2A). It should be noted that since HEK293 cells express TRPV4 at very low level the endogenous TRPV4 could also contribute into the observed responses. To study this translocation systematically, we developed a BRET assay between TRPV4-Venus and TRPV4-Rluc8 to measure the degree of TRPV4 channel aggregation. Using this assay, we found that GSK1016790A caused the concentration-dependent cytoplasmic aggregation of TRPV4 with an EC_50_ value of 31 nM. GSK1016790A (100 nM) produced a near maximal effect, so we used this concentration for the rest of the study (Figure 2B).

To confirm that GSK1016790A induced the translocation of TRPV4 from the plasma membrane, we assessed the ratio of BRET between TRPV4-Rluc8 and Kras-Venus, a plasma membrane marker and Rab11-Venus, a recycling endosome marker. At 30 min post-TRPV4 stimulation with GSK1016790A, the net BRET ratio between TRPV4-Rluc8 and Kras-Venus declined by (0.027 ± 0.011 fold, *p* < 0.01), while the BRET ratio between TRPV4-Rluc8 and Rab11-Venus increased by (0.028 ± 0.008 fold, *p* < 0.05). Together, these data showed that within 30 min post- TRPV4 stimulation with GSK1016790A, TRPV4 was internalized and transferred into recycling endosomes (Figure 2C).

**Figure 3:**
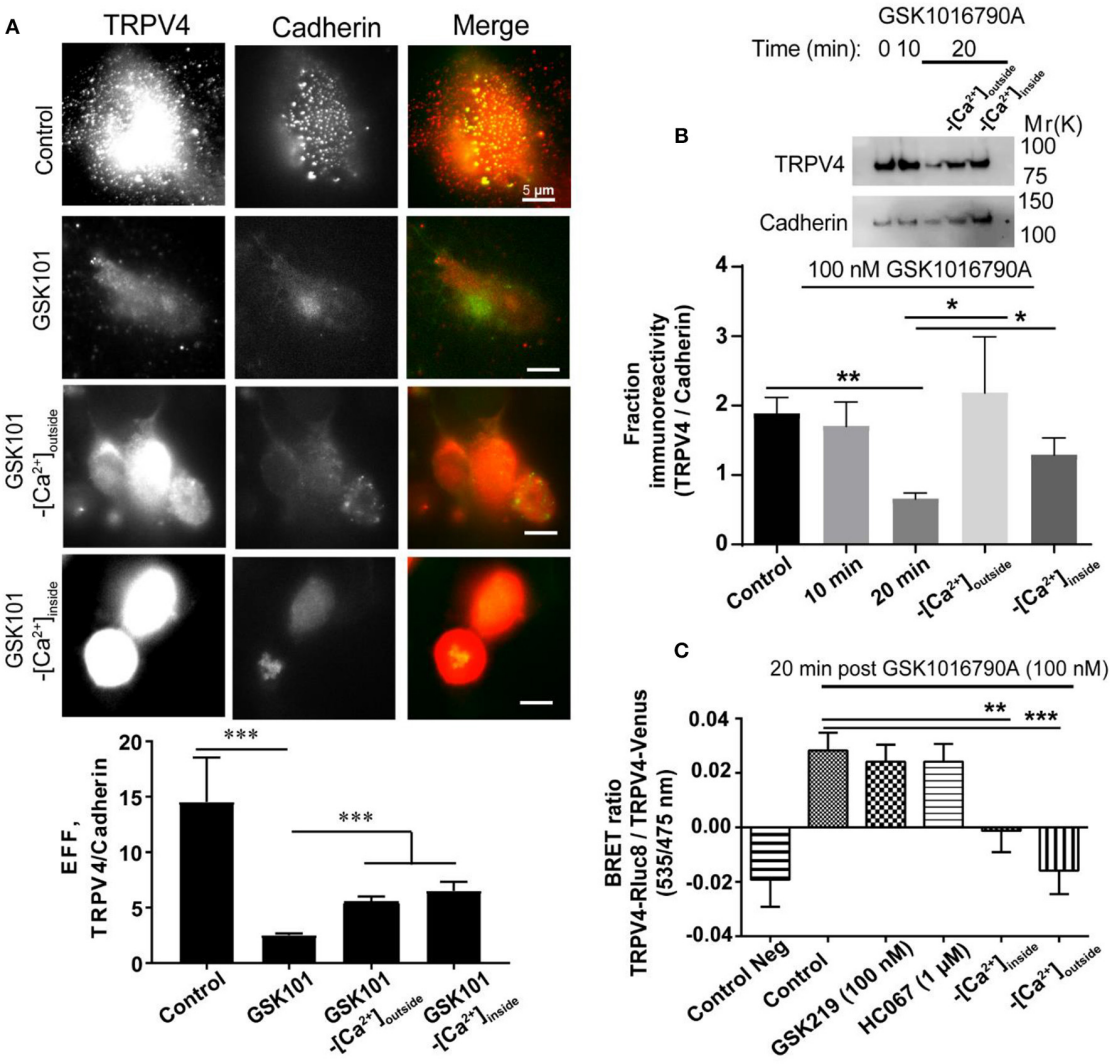
The internalization of TRPV4 post-GSK1016790A stimulation is Ca^2+^ dependent **(A)** TIRF microscopy and **(B)** Cell surface biotinylation assay in HUVECs showed that TRPV4 stimulation with GSK1016790A reduces the membrane density of TRPV4 channels while depletion of calcium from the stores or removal of the calcium from the buffer inhibits this internalization. **(C)** BRET assay in HEK293 cells expressing TRPV4-Rluc8 and TRPV4-Venus showed that depletion of calcium from the stores (-[Ca^2+^]_inside_) with 1 μM thapsigargin or removal of the calcium from the buffer (-[Ca^2+^]_outside_) reduces the aggregation of TRPV4 channels post-GSK1016790A stimulation while inhibition of TRPV4 with GSK2193874 (GSK219) and HC067047 (HC067) did not have any effect. Data are representative of at least four independent experiments. ^*^p < 0.05, ^**^p < 0.01, and ^***^p < 0.001.

First, we studied the effect of GSK1016790A on the localization of endogenous TRPV4 expressed in HUVECs. For this, we first used a cell surface biotinylation assay, and in agreement with a previous study (Jin et al., 2011), we found that the treatment of endothelial cells with an agonist for 20 min reduced the membrane expression of TRPV4 by (1.16 ± 0.30 fold, *P* < 0.01) (Figure 3A). We investigated the role of Ca^2+^ (influx or release) in endocytosis of TRPV4 post GSK1016790A treatment in HUVECs using both TIRF microscopy and cell surface biotinilation assay and using both approach found that depletion of Ca^2+^ from intracellular stores or removal of Ca^2+^ from the buffer reduced the endocytosis of TRPV4 post-GSK1016790A treatment (Figures 3A,B).

To identify whether TRPV4 internalization and aggregation is dependent on Ca^2+^ release from the intracellular stores or Ca^2+^ influx from the extracellular environment, we depleted intracellular Ca^2+^ stores by using thapsigargin or by removing Ca^2+^ from the buffer (extracellular space). Under both conditions, TRPV4 stimulation with GSK1016790A failed to induce cytoplasmic aggregation of TRPV4 channels, suggesting that this phenomenon is dependent on both Ca^2+^ release from intracellular stores as well as Ca^2+^ influx from the extracellular environment (Figure 3C). We used both GSK2193874 and HC067047, selective antagonists of TRPV4, to evaluate whether internalization is dependent on Ca^2+^ influx through TRPV4. We found that the removal of calcium from the buffer reduced the TRPV4 aggregation by (0.04 ± 0.01 fold, *p* < 0.001), and depletion of calcium from the stores reduced the aggregation by (0.03 ± 0.01 fold, *p* < 0.01), while the inhibition of TRPV4 did not have any effect (Figure 3B). However, it should be noted that 1 μM of HC067047 does not entirely block the calcium influx via TRPV4 (Figures 1A,B).

### Two Modes of Membrane Trafficking Present for TRPV4 Channels

To elucidate the early events following TRPV4 stimulation with GSK1016790A, we utilized live-cell TIRF microscopy to examine the dynamics of the TRPV4 channel translocation and internalization post-GSK1016790A stimulation. We used HEK293 cells stably expressing TRPV4 channels tagged with Venus on their C-terminus. With this approach, individual trafficking to and from the plasma membrane were easily observed as rapid, discrete increased in fluorescent intensity. We visualized individual TRPV4 channels in kymographs, in which a cross-section of the cell is depicted over time. Maximum intensity measurements from these events did not show any significant change within the first 2 min of stimulation. We found TRPV4 channels in the form of vesicular structures with two distinct characters. The first group were vesicles that had complete fusion to the plasma membrane, and their intensity did not change during the 1 min of imaging (Supplementary Video 2).

**Figure 4:**
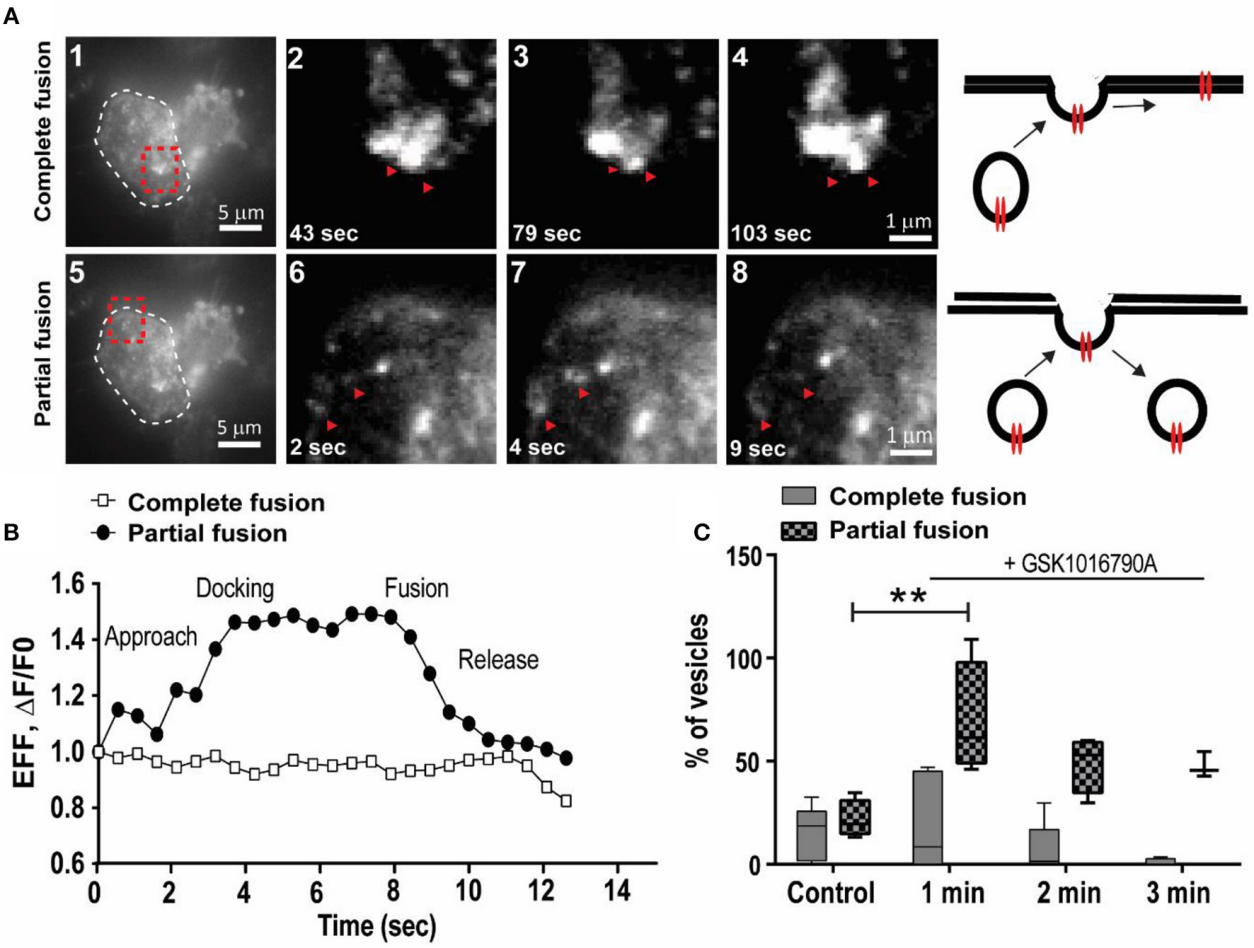
GSK1016790A induced the partial fusion of TRPV4 channels to the plasma membrane. **(A)** TIRF images show two modes of trafficking for TRPV4-Venus molecules, complete, and partial fusion (red arrows), cell border is labeled with white dashed line in a1 and a5 and selected regions (shown by red dashed line is magnified in a2–4 and a6–8. Images are selected from Supplementary Video 2. **(B)** Representative graphs from data depicted in **(A)** showing the dynamics of complete and partial fusion of TRPV4-Venus expressed in HEK293 cells. **(C)** During the first minute of applying GSK1016790A there was an increase in the density of TRPV4 channels with partial fusion. Data are representative of at least four independent experiments. ^**^P < 0.01, One-Way ANOVA, Dunnett's *post-hoc* test.

In comparison, the second group showed the characteristics of vesicles with partial fusion that, on average, had 8–20 s residence time at the plasma membrane (Supplementary Video 2), Figures 4A,B). We analyzed the effect of 100 nM GSK1016790A on dynamics of TRPV4 channel trafficking. In the control group, the density of vesicles with both partial and complete fusion reduced during the 3 min of imaging (Supplementary Video 2).

Addition of GSK1016790A did not change the density of TRPV4 vesicles undergoing complete fusion within the first 3 min post-stimulation while the density of TRPV4 channels with partial fusion increased by (47.71 ± 14.46 fold, *p* < 0.05) within the first minute (Supplementary Video 3) while the residence time for agonist treated vesicles decreased to < 8 s (Figure 4C). It is reported that raise in the pH could cause the fluorescent proteins to brighten (Han et al., 1999; Taraska et al., 2003). However, the increased in the partial fusion events here were only observed after the addition of GSK1016790A, hence it is a triggered event.

For the vesicles with partial fusion, because intensity at the insertion site between the consecutive events did not reach the background level, we hypothesized that the partial fusions of TRPV4 channels are indicative of kiss-and-run types of trafficking (Alabi and Tsien, 2013). To confirm this hypothesis, we targeted dynamin. Dynamin has been postulated to be important in the retrieval of exocytotic vesicles after kiss-and-run-exocytosis (Graham et al., 2002; Holroyd et al., 2002).

**Figure 5:**
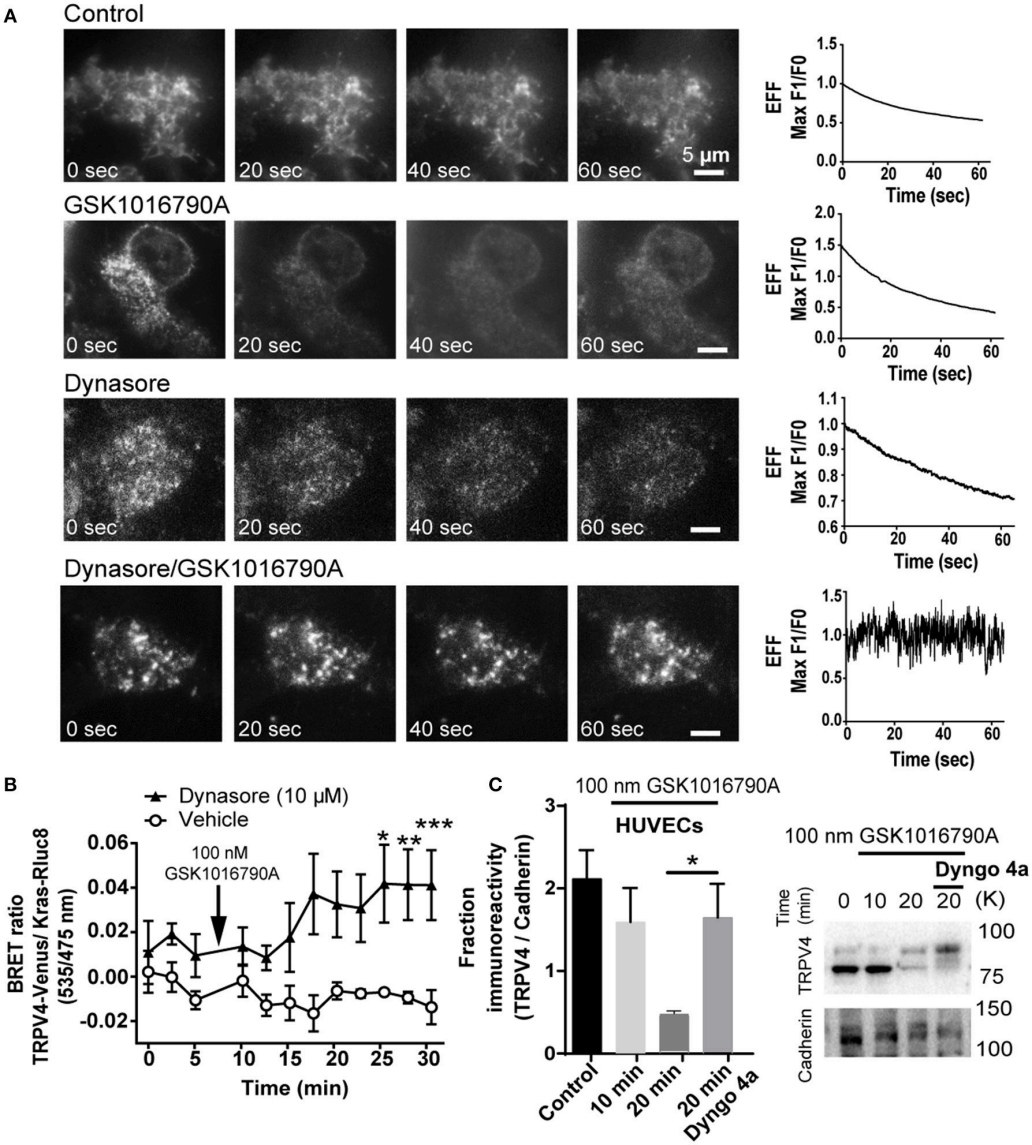
GSK1016790A induced partial fusion of TRPV4 is dynamin dependent **(A)** TIRF microscopy images showed that inhibition of dynamin blocks the internalization of TRPV4-Venus post-GSK1016790A stimulation while in control groups; untreated, GSK1016790A or Dynasore only treated cells, there is a decrease in the intensity of the TRPV4-Venus fluorescent signal. Graphs represent the evanescence field intensity of the selected cell across the time. **(B)** BRET data are showing that inhibition of dynamin with 10 μM of dynasore increased the net BRET ratio between TRPV4 and membrane marker Kras. **(C)** Cell surface biotinylation assay is showing that inhibition of dynamin with 30 μM Dyngo4a increased the membrane expression of TRPV4 in HUVECs. TRPV4 on the membrane is expressed in two forms of glycosylated and non-glycosylated. The higher molecular weight band represents the glycosylated form (Lamandé et al., 2011). The graph in **(C)** represents the average intensity of both bands normalized to the Cadherin, the membrane marker. Data are representative of four independent experiments, and ^*^p < 0.05, ^**^p < 0.01, and ^***^p < 0.01.

Pre-treatment of HEK293-TRPV4-Venus cells with dynamin inhibitor 30 min before TRPV4 agonist stimulation caused the progressive increase in the density of TRPV4 at the TIRFM field of view that was absent in non-stimulated cells or cells that were treated with GSK1016790A or Dynasore as control groups (Figure 5A) (Supplementary Videos 3–6).

Further, we repeated this experiment with BRET assay and measured the change in the ratio of BRET signal between TRPV4-Venus and Kras-Rluc8. In agreement with the TIRFM data, we found that inhibition of dynamin increases the BRET signal between TRPV4 and Kras confirming that vesicles with partial fusion are dependent on dynamin GTPase activity (Figure 5B). To confirm the BRET data with TRPV4-HEK293 cells, we used a cell surface biotinylation assay with endothelial cells (HUVECs) and found that like HEK293 cells, inhibition of dynamin inhibits the endocytosis of TRPV4 channels post GSK1016790A stimulation by (1.2 ± 0.3 fold, *p* < 0.05) (Figure 5C), and therefore we concluded that GSK1016790A increased the kiss-and-run trafficking of TRPV4 channels at the cell surface.

### Endocytosis of TRPV4 Channels Following TRPV4 Stimulation With GSK1016790A Is Controlled via PKC and PI3K Signaling

We next investigated which signaling molecule is required for the TRPV4 channel partial fusion and endocytosis following TRPV4 stimulation with GSK1016790A. Rho GTPases play an important role in the regulation of different endocytic pathways. For example Rac1, CDC42, and RhoA have been postulated to regulate clathrin-mediated endocytosis (Qualmann and Mellor, 2003). To investigate the involvement of small G protein RhoA, CDC42, Rac1, and Gαq in GSK1016790A induced endocytosis of TRPV4, in HEK293 cells stably expressing TRPV4-Rluc8 we transiently transfected either wild-type: RhoA, CDC42, Gαq or Rac1, or the dominant negative form: (T19N) RhoA, (T17N) CDC42, (Q209L/D277N) Gαq or (T17N) Rac1 with Kras-Rluc8 as a membrane marker. Next, we measured the changes in the ratio of BRET signal between the TRPV4-Venus and Kras-Rluc8 to assess the regulatory role of Rho GTPases on GSK1016790A mediated endocytosis of TRPV4.

**Figure 6:**
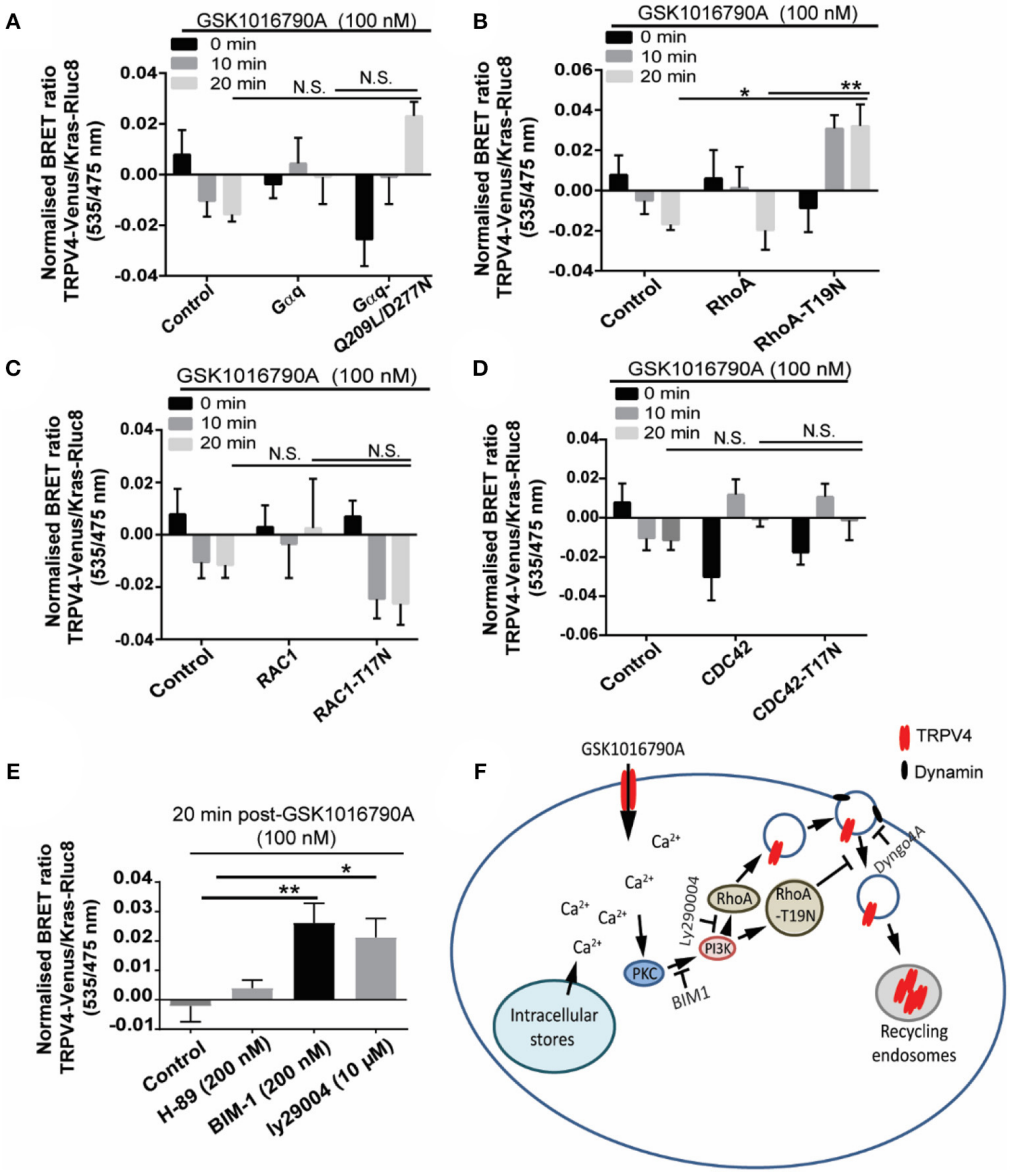
GSK1016790A induced TRPV4 endocytosis is controlled via PKC/PI3K and RhoA signaling pathways. **(A–D)** BRET assay showed that over-expression of dominant negative RhoA increased the BRET ratio between TRPV4 and Kras after 20 min treatment with GSK1016790A while there was no difference between dominant negative and wild-type of RAC1, CDC42, and Gαq. **(E)** BRET assay showed that inhibition of PI3K with 10 μM of Ly294002 (PI3K inhibitor) and 200 nM of BIM1 (PKC inhibitor) increased the net BRET ratio between TRPV4 and Kras after 20 min treatment with GSK1016790A while inhibition of PKA using 200 nM of H-89 did not have any effect. **(F)** The cartoon is showing the proposed signaling pathway controlling the internalization of TRPV4 post-GSK1016790A stimulation. Data are representative of mean ± SEM of four independent experiments and ^*^p < 0.05, and ^**^p < 0.01.

Using this approach, we found GSK1016790A-dependent increase in the net BRET signal between TRPV4 and Kras in presence of dominant negative RhoA (T19N) that was significantly higher than the cells transfected with the wild-type RhoA (*p* < 0.01) and non-transfected control group (*p* < 0.05), 20 min post TRPV4 stimulation with GSK1016790A (Figures 6A–D) while no significant effect observed between TRPV4 and Kras BRET signal between the wild-type and dominant negative form of other GTPases, CDC42, Rac1, and Gαq and non-transfected control group (Van der Zee et al., 2004).

Because of the role of calcium in trafficking of TRPV4, we hypothesized that calcium influx should trigger the signaling pathway that leads to the partial fusion and endocytosis of TRPV4. Ca^2+^ is reported to activate Ca^2+^ dependent PKC isoforms (PKCalpha, beta1, beta2, and gamma), and in return, PKC phosphorylation leads to the activation of different TRP channels including TRPV4 (Van der Zee et al., 2004; Peng et al., 2010; Ghigo et al., 2017). Here, pre-treatment of HEK293 cells stably expressing TRPV4 with 100 nM of BIM1 [selective PKC inhibitor (Toullec et al., 1991)] blocked the TRPV4 channel endocytosis post-GSK1016790A stimulation by (0.03 ± 0.005 fold, *p* < 0.01) fold. In contrast, pre-treatment with PKA and PKG inhibitors did not have any effect of on the ratio of BRET signal between TRPV4 and Kras. This result is consistent with our previous observation that PKA and PKG inhibitors do not affect the elevation of [Ca^2+^]_i_ following TRPV4 stimulation with GSK1016790A (Baratchi et al., 2016). PKC is reported to trigger the activation and recruitment of PI3K to the plasma membrane (Ziemba et al., 2016). PI3K can mediate intracellular vesicular trafficking of different receptors.

Here, pre-treatment with 10 μM of Ly294002 [potent PI3K inhibitor (Vlahos et al., 1994)] blocked the TRPV4 channel endocytosis following TRPV4 stimulation with GSK1016790A by increasing the Bret signal between the TRPV4 and Kras from −0.005 ± 0.004 to 0.019 ± 0.007, (0.02 ± 0.009 fold, *p* < 0.05) (Figure 6E). It should be noted that the downstream effector molecule of PI3K, PKB did not affect the TRPV4 response to GSK1016790A.

## Discussion

Endosomal delivery of transmembrane receptors to the plasma membrane is necessary for many physiological processes such as endothelial cell-mediated dilatation of small blood vessels in response to blood flow (Sorkin and von Zastrow, 2009). The contribution of membrane trafficking/endocytosis of Ca^2+^ ion channels in maintaining Ca^2+^ homeostasis has been defined before (Xue et al., 2012). However, the mechanism and kinetics by which exocytic/endocytic vesicles fuse and deliver ion channels to and from the plasma membrane remained poorly defined.

There is a growing appreciation that local increased in [Ca^2+^]_i_ are important for the regulation of vascular function. TRPV4 is a candidate mechanoreceptor that is widely expressed in the endothelium. Ca^2+^ influx through TRPV4 is an important source of the local Ca^2+^ increase in the endothelium (Han et al., 1999). It has also been shown that the cooperative opening of as few as three clustered TRPV4 channels per endothelial cell, caused maximal vasodilation through the activation of intermediate- and small- conductance Ca^2+^ sensitive potassium channels (Sonkusare et al., 2012, 2014). We have recently shown that physiological shear stress increased the trafficking of TRPV4 channels to the plasma membrane which sensitized endothelial cell responses to a selective TRPV4 agonist, GSK1016790A (Baratchi et al., 2016).

GSK1016790A is the potent and selective agonist of TRPV4 that has been widely used as a valuable tool to study the physiological properties of TRPV4 (Pankey et al., 2014; Xu et al., 2016; Tian et al., 2017). Activation of TRPV4 with GSK1016790A has been reported to inhibit TNF-α-induced monocyte adhesion to human endothelial cells, and oral administration of GSK1016790A reduces atherosclerotic plaque formation (Sullivan et al., 2012). However, the nature of TRPV4 channel responses to this drug is not very well understood.

Here, we investigated the effect of small molecule GSK1016790A on vesicular fusion events mediating the trafficking of TRPV4 channels in HEK293 cells as well as primary endothelial cells. Further, we elucidate the signaling pathway that controls TRPV4 channel trafficking in response to GSK1016790A (Figure 6F).

First, we showed that GSK1016790A caused early and rapid activation of TRPV4 channels, leading to an increase in [Ca^2+^]_i_. Second, we showed that TRPV4 channels could be delivered to the cell surface via two modes of exocytosis: complete fusion and partial fusion. Within few seconds of TRPV4 stimulation with GSK1016790A, the number of vesicles with partial fusion increased, while the number of vesicles with complete fusion decreased. This event coincided with peak Ca^2+^ influx into the cytoplasm and localized transient increase of Ca^2+^ close to the plasma membrane. Previously it was shown that quiescent TRPV4 channels were recruited to be activated by GSK1016790A (Sullivan et al., 2012). Further to that our results suggests that localized influx of Ca^2+^ into the cytoplasm is regulated via the partial fusion of TRPV4 channels to the cell membrane. Third, we show that activation by GSK1016790A leads to subsequent endocytosis of TRPV4 channels into the cytoplasm, where they accumulate into the recycling endosomes. Therefore, the early, rapid activation of the channel is likely to reflect the activity of newly recruited channels to the plasma membrane and the subsequent decrease in the membrane expression of the TRPV4 channels that is reported by Min Jin et al. (2011) and us here is reflecting the endocytosis of TRPV4 channels into the recycling endosomes. It should be noted that we have not observed any effect on the membrane expression of TRPV4 post stimulation with 4 alpha PDD, another TRPV4 agonist showing that the observed responses of TRPV4 to GSK1016790A are agonist specific.

Trafficking of vesicles to the plasma membrane is a highly regulated process that is counterbalanced with endocytosis to ensure constant turnover of membrane contents. Membrane trafficking and exocytosis uses a cascade of protein interactions to ensure efficient delivery of their cargo to the plasma membrane (Planells-Cases and Ferrer-Montiel, 2007).

Endocytosis of membrane receptors is regulated by a Clathrin-dependent pathway that directs endocytosis vesicles into their destination (Royle and Murrell-Lagnado, 2003). Triggering of SNARE-mediated exocytosis of recycling vesicles is dependent on an increase in [Ca^2+^]_i_ (Planells-Cases and Ferrer-Montiel, 2007). Ca^2+^ release from the intracellular stores is described to precisely control exocytosis and endocytosis (Royle and Murrell-Lagnado, 2003; Low et al., 2010). Based on our observations that removal of Ca^2+^ from the extracellular medium and depletion of the Ca^2+^ from the intracellular stores controls endocytosis of TRPV4 post-GSK1016790A stimulation, we conclude that TRPV4 internalization is controlled by both cytosolic Ca^2+^ microdomains as well as influx of Ca^2+^ from the intracellular stores.

In this study, we have also provided evidence that GSK1016790A induced endocytosis of TRPV4 is regulated via PI3K and PKC. Direct activation of PKC with β-phorbol 12-myristate 13-acetate (PMA) controls trafficking Cav1.2 in a mouse atrium cell line (HL-1) (Peng et al., 2010) and neuronal NMDA receptor in hippocampal slices (Yan et al., 2011). Furthermore, an isoform of PKC, PKCδ is reported to control the activity of store-operated Ca^2+^ entry in airway smooth muscle cells (Gao et al., 2012). Therefore, calcium influx via TRPV4 and downstream activation of PKC could also lead to the activation of other molecules that their activity is PKC dependent.

In distal nephrons (Mamenko et al., 2013) and endothelial cells (Baratchi et al., 2016), PKA has been shown to control the trafficking of TRPV4 to the apical membrane, while the PKC-dependent pathway has been postulated to stimulate the activity of TRPV4 (Mamenko et al., 2013). Further, is shown that activation of PKC with PMA leads to the phosphorylation of TRPV4 on serine 824 that is prevented with PKC inhibitor (Peng et al., 2010). While not within the scope of this paper, our data here is consistent with the hypothesis that phosphorylation on as yet unrecognized PKC site controls the trafficking of TRPV4 after stimulation with GSK1016790A.

PI3K plays a regulatory role in clathrin-dependent endocytosis of different types of membrane proteins, including renal outer medullary potassium channels (Cheng and Huang, 2011), potassium channel Kv7.1 in endothelium (Andersen et al., 2013), voltage-dependent calcium channels (Viard et al., 2004), and thermosensor TRPV1 (Stein et al., 2006). Here, we also found that PI3K plays an essential role in the endocytosis of TRPV4 following GSK1016790A stimulation as inhibition of PI3K increased the membrane expression of TRPV4 post GSK1016790A stimulation that we attribute to the regulatory effect of TRPV4 endocytosis. Rho family GTPases play a crucial role in tethering and fusion of the exocytic vesicles as well as endocytosis (de Curtis and Meldolesi, 2012). In this study, we found that overexpression of dominant negative RhoA blocks the internalization of TRPV4 channels post GSK1016790A stimulation and leads to the increase in the membrane expression of TRPV4.

Overnight endothelial TRPV4 stimulation with GSK1016790A has been shown to protect the endothelium from atherosclerotic plaque formation at the regions of arteries that blood flow is disturbed (Sullivan et al., 2012). The mechanotransduction of blood flow in the endothelium through adherens junctions plays an important role in the development of atherosclerosis (Gulino-Debrac, 2013; Dorland and Huveneers, 2016). On the membrane, TRPV4 is found in adherens junctions and has been reported to interact with the cadherin complex through ß-catenin (Janssen et al., 2011; Baratchi et al., 2017b). Here, we showed that the endocytosis of TRPV4 channels in the endothelial cell after agonist stimulation is associated with the internalization of the adherens junction protein, VE-cadherin. Adherens junctions maintain the membrane permeability function and form a complex with another cadherin in the neighboring cells. This process raises the possibility that the interaction of TRPV4 with adherens junction proteins and force-dependent Ca^2+^ influx may play a regulatory function in the mechanotransduction of blood flow and the development of atherosclerosis in regions where blood flow is disturbed. Our finding suggests that the vasoprotective effect of GSK1016790A on endothelium could be attributed at least in part to its regulatory role in controlling the basolateral expression of cadherins and β-catenin.

## Author Contributions

SB and PM: designed the experiments; SB, PK, WD, AL, PV, and KE: performed the experiments; SB, PK, WD, KK, and PT: analyzed the data; SB and PM: wrote the manuscript.

### Conflict of Interest Statement

The authors declare that the research was conducted in the absence of any commercial or financial relationships that could be construed as a potential conflict of interest.

## Supplementary Material

Supplementary Video 1 Calcium imaging experiment showing the effect of GSK1016790A (GSK101) on [Ca^2+^]_i_ level of HUVECs.

Supplementary Video 2 TIRF imaging shows two modes of trafficking for TRPV4-Venus molecules, complete and partial fusion (black arrows).

Supplementary Video 3 TIRF imaging of HEK293-TRPV4-Venus cells, control group shows that there is a decrease in the intensity of the TRPV4-Venus fluorescent signal.

Supplementary Video 4 TIRF imaging of HEK293-TRPV4-Venus cells treated with GSK1016790A (GSK101) shows that there is a decrease in the intensity of the TRPV4-Venus fluorescent signal.

Supplementary Video 5 TIRF imaging of HEK293-TRPV4-Venus cells treated with dynasore only shows that there is a decrease in the intensity of the TRPV4-Venus fluorescent signal.

Supplementary Video 6 TIRF imaging of HEK293-TRPV4-Venus cells pre-treated with dynasore for 30 min followed by treatment with 100 nM of GSK1016790A (GSK101). White arrows show TRPV4-venus channels that are recruited to the plasma membrane after inhibition of dynamin and stimulation of TRPV4 with GSK101.
