# Supplementary material for: The TRPV4 Agonist GSK1016790A Regulates the Membrane Expression of TRPV4 Channels
Source: Front Pharmacol. 2019 Jan 23;10:6. doi: 10.3389/fphar.2019.00006 (PMC6351496; doi:10.3389/fphar.2019.00006)
Supplement: Supplementary file 1 [file Image_1.pdf]

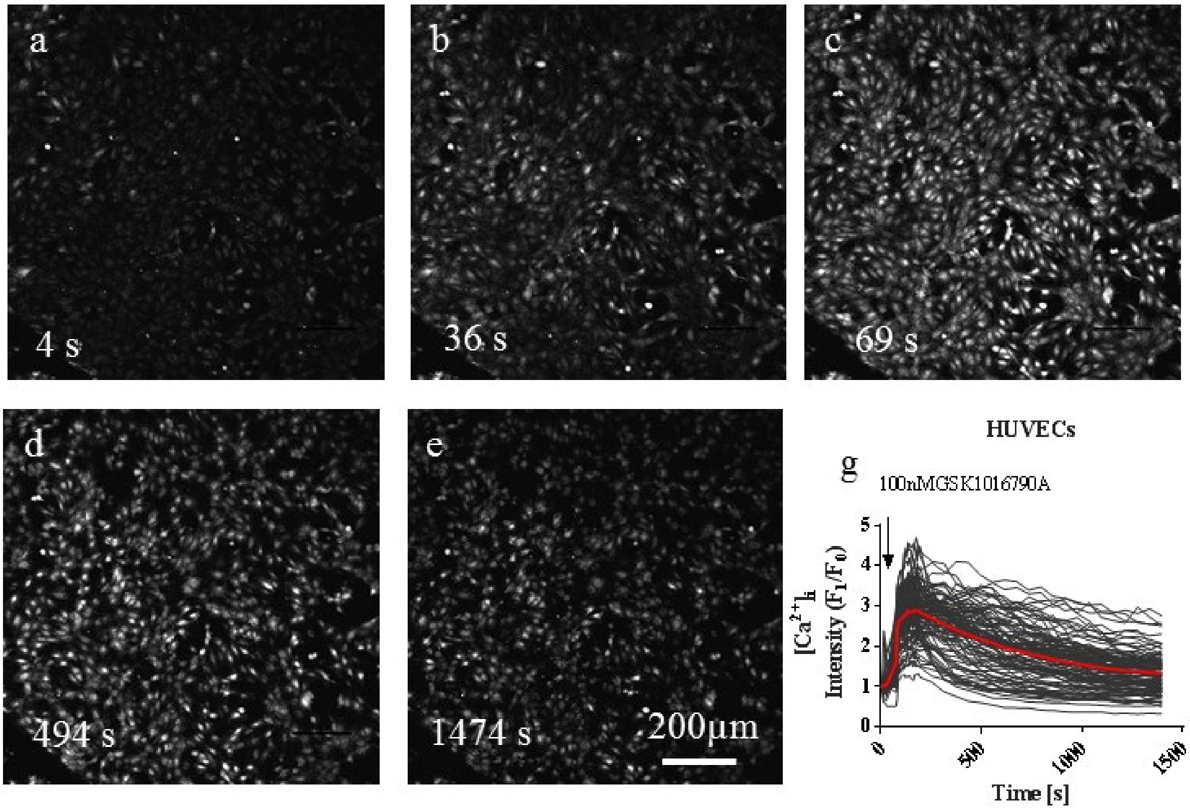

Supplementary 1. Calcium imaging experiment showing the effect of GSK101 on $[Ca^{2+}]_i$ level of HUVECs. a-e Representative images selected from Movie 1. g. Black lines in graph represent single cell responses, and red line represents average cellular responses.
